# Ablation of sphingosine kinase 2 suppresses fatty liver-associated hepatocellular carcinoma via downregulation of ceramide transfer protein

**DOI:** 10.1038/s41389-022-00444-0

**Published:** 2022-11-04

**Authors:** Xin Tracy Liu, Long Hoa Chung, Da Liu, Jinbiao Chen, Yu Huang, Jonathan D. Teo, Xingxing Daisy Han, Yinan Zhao, Fiona H. X. Guan, Collin Tran, Jun Yup Lee, Timothy A. Couttas, Ken Liu, Geoffery W. McCaughan, Mark D. Gorrell, Anthony S. Don, Shubiao Zhang, Yanfei Qi

**Affiliations:** 1 Centenary Institute, The University of Sydney, Sydney, NSW Australia; 2 School of Medical Sciences and Charles Perkins Centre, Faculty of Medicine and Health, University of Sydney, Sydney, NSW Australia; 3 Key Laboratory of Biotechnology and Bioresources Utilization of Ministry of Education, Dalian Minzu University, Dalian, Liaoning China; 4 AW Morrow Gastroenterology and Liver Centre, Royal Prince Alfred Hospital, Sydney Local Health District, Sydney, NSW Australia; 5 Brain and Mind Centre, Faculty of Medicine and Health, University of Sydney, Sydney, NSW Australia

**Keywords:** Cancer metabolism, Lipid signalling

## Abstract

Hepatocellular carcinoma (HCC) accounts for 90% of primary liver cancer, the third leading cause of cancer-associated death worldwide. With the increasing prevalence of metabolic conditions, non-alcoholic fatty liver disease (NAFLD) is emerging as the fastest-growing HCC risk factor, and it imposes an additional layer of difficulty in HCC management. Dysregulated hepatic lipids are generally believed to constitute a deleterious environment cultivating the development of NAFLD-associated HCC. However, exactly which lipids or lipid regulators drive this process remains elusive. We report herein that sphingosine kinase 2 (SphK2), a key sphingolipid metabolic enzyme, plays a critical role in NAFLD-associated HCC. Ablation of *Sphk2* suppressed HCC development in NAFLD livers via inhibition of hepatocyte proliferation both in vivo and in vitro. Mechanistically, SphK2 deficiency led to downregulation of ceramide transfer protein (CERT) that, in turn, decreased the ratio of pro-cancer sphingomyelin (SM) to anti-cancer ceramide. Overexpression of CERT restored hepatocyte proliferation, colony growth and cell cycle progression. In conclusion, the current study demonstrates that SphK2 is an essential lipid regulator in NAFLD-associated HCC, providing experimental evidence to support clinical trials of SphK2 inhibitors as systemic therapies against HCC.

## Introduction

Primary liver cancer was estimated to result in 8.3% of all cancer-related deaths worldwide in 2020, making it the third leading cause following lung and colorectal cancer [1]. Hepatocellular carcinoma (HCC) accounts for >90% of primary liver cancer [2]. In recent years, non-alcoholic fatty liver disease (NAFLD) is emerging as the fastest-growing etiology of HCC in developed countries [2]. In HCC of all causes, cirrhosis is the most prevalent pathological event [3]. However, approximately 40% of NAFLD-associated HCC (NAFLD-HCC) can develop from non-cirrhotic livers [4], suggesting that NAFLD-HCC may represent a unique pathogenic route. In NAFLD-HCC, hepatic lipid dysregulation is believed to constitute a deleterious environment that cultivates cancer initiation and progression. However, the lipid risk factors in this condition have not yet been adequately explored [5]. So far, the management of HCC remains challenging. NAFLD creates an additional layer of difficulty in the systemic treatment [6], and, worse still, there is no FDA-approved drug for NAFLD [7]. As such, understanding the lipid metabolic causes of NAFLD-HCC is a fundamental step in developing effective treatment and a key topic in liver cancer research.

Sphingolipids are essential lipids that act as cell membrane constituents and signaling molecules [8] and have been implicated in cancer development [9, 10]. Ceramide is the central metabolite in the sphingolipid metabolic network [8–10]. Once synthesized in the endoplasmic reticulum, the majority of ceramide serves as the substrate for the synthesis of more complex sphingolipids, mainly sphingomyelin (SM) [11]. In the ceramide-to-SM conversion, ceramide is delivered to the *trans*-Golgi by its unique intracellular transporter, ceramide transfer protein (CERT) [12], which enables its access to sphingomyelin synthase 1 and 2 (SMS1 and SMS2) for SM production [13, 14]. SM is subsequently distributed to the plasma membrane or other subcellular membrane compartments [15], where it can be hydrolyzed back to ceramide by neutral sphingomyelinase (nSMase) or acid SMase (aSMase) [16, 17]. The balance between SM and ceramide is a critical determinant of cell fate. SM is considered a pro-cancer factor, promoting cell survival, proliferation, and migration [14, 18–20], and it is increased in human HCC compared with para-tumorous tissues [21–23]. In contrast, ceramide has been well characterized as an anti-cancer factor, inducing apoptosis and cell cycle arrest [10, 24], and it is decreased in human HCC compared with adjacent non-tumorous tissues [21–23]. Therefore, efforts to understand and modulate SM/ceramide homeostasis may provide a new approach to HCC management.

Sphingolipid levels are determined by their catabolism, where sphingosine kinase (SphK) is the rate-limiting enzyme [8]. In brief, ceramide is hydrolyzed into sphingosine and a free fatty acid (FFA), followed by SphK-mediated phosphorylation of sphingosine to form sphingosine 1-phosphate (S1P), and then hydrolysis of S1P into non-sphingolipid products [8, 9]. There are two mammalian isoforms of SphK, denoted SphK1 and SphK2. SphK2 is the predominant isoform in the liver, contributing to 90% of total SphK activity [25]. Only a few studies have investigated the role of SphK2 in HCC. Administration of the selective SphK2 inhibitor ABC294640 profoundly suppresses the growth of HepG2 or SK-HEP-1 HCC xenografts [26]. Knockdown or inhibition of SphK2 sensitizes HCC cells to the chemotherapeutic agent regorafenib in vitro [27]. These all suggest a pro-cancer role of SphK2 in HCC. In addition, ABC294640 has been tested for HCC treatment in a Phase I clinical trial (ClinicalTrials.gov Identifier: NCT01488513), was planned to be used as a monotherapy for advanced HCC in a Phase II study (NCT02939807), and is being tested for intrahepatic cholangiocarcinoma (NCT03377179). Surprisingly, SphK2 has never been studied in any primary liver cancer models in vivo. Furthermore, we and others have identified SphK2 as a critical regulator of NAFLD and hepatic insulin resistance in diet-induced obese mice [28, 29]. Therefore, investigating the role of SphK2 in the development of NAFLD-HCC is important.

In this study, we examined the role of SphK2 in NAFLD-HCC using *Sphk2* knockout (KO) mice on a high-fat, high-sugar diet (HFHSD). In addition to liver tumor incidence, we examined immune cell infiltration, fibrosis and cell proliferation in mouse livers upon ablation of *Sphk2*. We also assessed the anti-cancer effects of SphK2 deficiency on cell viability, clonogenicity and cell cycle in hepatic cells exposed to a high-fat environment. Through a near complete profiling of the lipidome in mouse livers, we identified that deletion of *Sphk2* disrupted the balance between SM and ceramide via downregulation of CERT. We also visualized SM and ceramide levels in human HCC specimens using mass spectrometry imaging. We further showed that downregulation of CERT was responsible for the tumor-suppressive effects of SphK2 deficiency. Collectively, our study revealed a previously unknown link between SphK2 and CERT in sphingolipid homeostasis and a critical role of SphK2 in NAFLD-HCC.

## Results

### Ablation of *Sphk2* suppresses HCC development in diet-induced obese mice

**Fig. 1:**
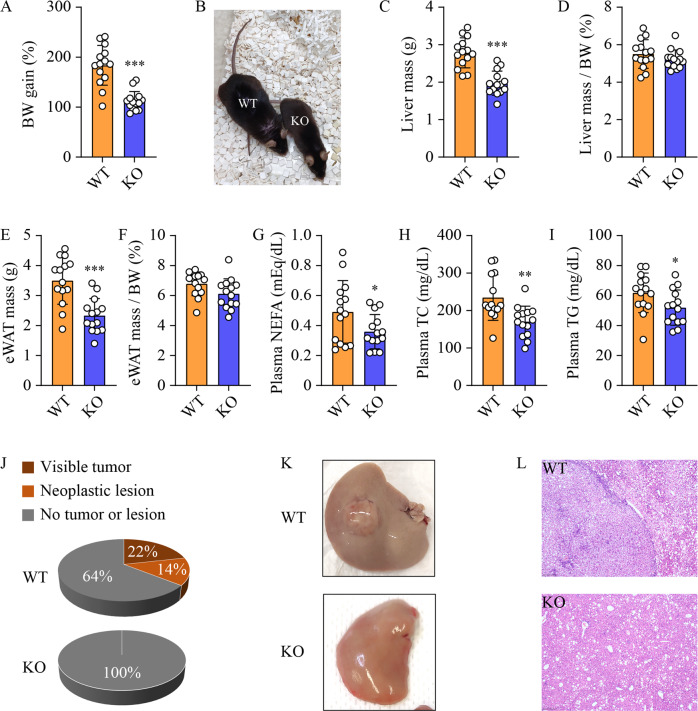
*Sphk2*-KO suppresses liver cancer development in HFHSD-fed mice. Wild-type (WT) and *Sphk2* knockout (KO) mice were fed with a high-fat, high-sugar diet (HFHSD) for 46 weeks. **A** body weight (BW) gain, **B** Photos of WT and KO littermates showing different BW gain, **C** liver mass, **D** % of liver mass/BW, **E** epididymal white adipose tissue (eWAT) mass, **F** % of eWAT/BW, **G** plasma non-esterified fatty acids (NEFA), **H** plasma total cholesterol (TC) and **I** plasma triglyceride (TG). **J** Tumor incidence. 3 of 14 WT developed visible tumors and an additional 2 of 14 WT mice developed neoplastic lesions in the liver, while 0 of 14 KO mice developed neither liver tumors nor lesions. **K** Macroscopic images of visible liver tumor. **L** Microscopic images of the neoplastic lesion following H&E staining, scale bar = 200 µm. Data are expressed as mean ± SD. *n* = 14. *p < 0.05; **p < 0.01; ***p < 0.001; versus WT.

The Cancer Genome Atlas (TCGA) data analyses showed that the hepatic *SPHK2* level was significantly increased in HCC as compared with normal livers, and it was further increased in extremely obese HCC patients (Suppl Fig. 1). This suggests an association of SphK2 with NAFLD-HCC, as NAFLD is highly prevalent in obese subjects [30]. To examine the role of SphK2 in the development of NAFLD-HCC, we fed wild-type (WT) and *Sphk2*-KO mice an HFHSD for 46 weeks in the absence of any chemical carcinogens. *Sphk2*-KO mice exhibited significantly lower body weight gain than their WT littermates (Fig. 1A, B). The liver mass and epididymal white adipose tissue (eWAT) mass were also lower in *Sphk2*-KO mice (Fig. 1C, E). However, the percentages of liver mass and eWAT mass to body weight were indistinguishable between the two genotypes (Fig. 1D, F). The levels of plasma non-esterified fatty acid (NEFA), total cholesterol (TC) and triglyceride (TG) were decreased in *Sphk2*-KO mice (Fig. 1G–I). Visible liver tumors developed in 3 of 14 WT mice, and neoplastic lesions were found in the liver of an additional 2 WT mice by microscopic examination (Fig. 1J–L). In contrast, neither visible tumors nor neoplastic lesions were identified in the equivalent number of *Sphk2*-KO mice (Fig. 1J–L).

### Ablation of *Sphk2* mitigates HCC-promoting pathological changes in non-tumorous liver tissues

**Fig. 2:**
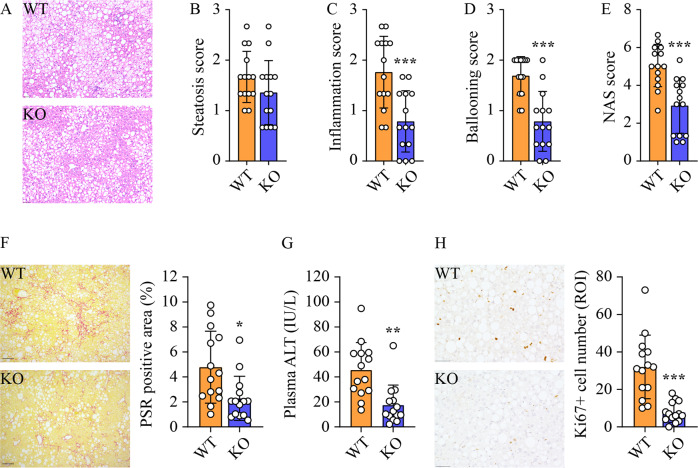
*Sphk2*-KO mitigates HCC-promoting pathological changes in non-tumorous liver tissues. Wild-type (WT) and *Sphk2* knockout (KO) mice were fed with a high-fat, high-sugar diet (HFHSD) for 46 weeks. **A** H&E staining of non-tumorous liver tissues. **B** Steatosis, **C** inflammation and **D** ballooning were scored based on the H&E staining. **E** NAFLD activity score (NAS) was the unweighted sum of steatosis, inflammation, and ballooning scores. **F** Hepatic fibrosis in non-tumorous tissue was visualized and quantified using Picro Sirius Red (PSR) staining. **G** Plasma ALT level. **H** Cell proliferation in non-tumorous tissue of WT and KO livers was stained and quantified by Ki67 immunohistochemistry. Scale bar = 50 µm. Data are expressed as mean ± SD. *n* = 14. *p < 0.05; **p < 0.01; ***p < 0.001; versus WT.

Extensive efforts have been made to elucidate cellular and histological disparities between tumorous and non-tumorous tissue in HCC, but the microenvironmental factors that determine HCC outcomes in non-tumorous tissues are understudied. In the following work, we mainly focused on analyzing the differences in non-tumorous liver tissues of WT and *Sphk2*-KO mice in order to understand why *Sphk2*-KO suppressed NAFLD-HCC. Comparable levels of steatosis were found in the parenchyma of WT and *Sphk2*-KO livers using hematoxylin and eosin (H&E) staining (Fig. 2A, B). However, *Sphk2*-KO significantly ameliorated immune cell infiltration, hepatocyte ballooning, NAFLD activity score (NAS) and hepatic fibrosis in non-tumorous tissues (Fig. 2C–F), which are classic hallmarks of pro-carcinogenic injury in NAFLD-HCC. In line with this, plasma alanine aminotransferase (ALT) activity was reduced by 66% in *Sphk2*-KO mice (Fig. 2G). In addition, the percentage of cells positive for proliferation marker Ki67 was decreased by 3.4-fold in *Sphk2*-KO livers (Fig. 2H), suggesting that SphK2 deficiency might exhibit anti-NAFLD-HCC effects at the cellular level.

### Anti-cancer effects of SphK2 deficiency in FFA-treated hepatic cells

**Fig. 3:**
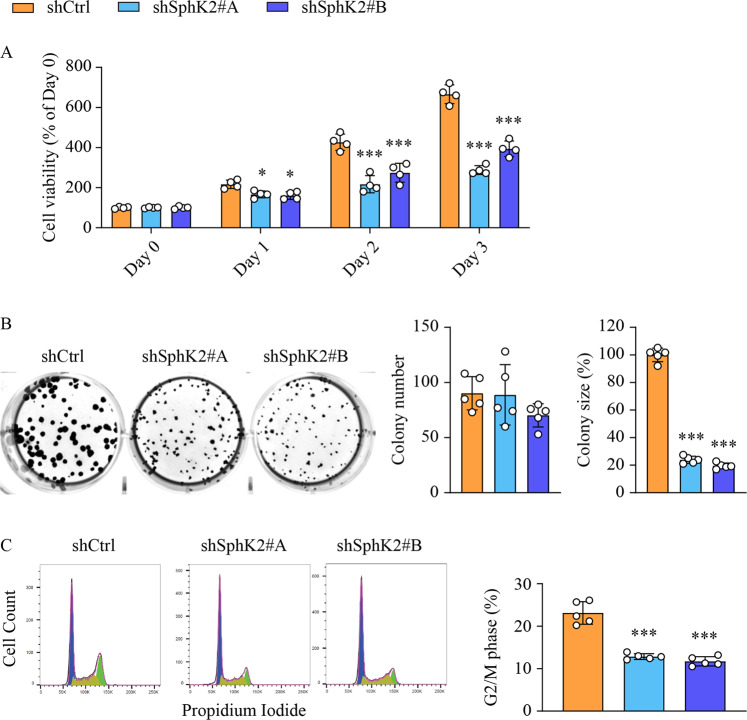
The anti-cancer properties of SphK2 deficiency in FFA-treated hepatic cells. Huh7 hepatic cells were transduced with lentiviral-based short hairpin RNA (shRNA) to knock down SphK2 and then treated with a combination of free fatty acids (FFA, 200 µM palmitate + 400 µM oleate). **A** Cell viability was determined by MTS assay in cells treated with FFA for the indicated times; *n* = 4. **B** Colony formation assay was performed over ten days of cell culture in the presence of FFA. The number and size of colonies were quantified; *n* = 5. **C** Cell cycle phases were examined in cells treated with FFA for 24 h, using flow cytometry with propidium iodide staining. G_0_/G_1_, S, and G_2_/M phase populations were estimated using FlowJo software and are indicated in blue, yellow and green in histograms, respectively. The percentage of the G_2_/M phase, indicative of cell cycle progression, was analyzed, *n* = 5. Data are expressed as mean ± SD. *p < 0.05; ***p < 0.001; versus shCtrl.

We next examined whether SphK2 deficiency led to anti-cancer effects in hepatocytes. To simulate a high-fat environment in vitro, we treated Huh7 cells with a combination of palmitate and oleate. As expected, viable cell number doubled approximately every 24 h in the control group (shCtrl) over the three days of FFA treatment, whereas cell proliferation was impaired by shRNA-mediated knockdown of SphK2 (shSphK2, Fig. 3A). In the absence of FFA treatment, viable cell number increased at a lower rate, and SphK2 knockdown had minimal effects on cell proliferation (Suppl Fig. 2). Subsequently, we examined the clonogenicity of FFA-treated cells. The colony number was comparable between control and SphK2 knockdown cells, whereas colony size was decreased by 78–84% in SphK2 knockdown cells (Fig. 3B). This result also implies an anti-proliferative effect of SphK2 deficiency in a high-fat environment. To further this notion, we analyzed the cell cycle using flow cytometry. Knockdown of SphK2 significantly suppressed cell cycle progression to the G2/M phase (Fig. 3C).

### Ablation of *Sphk2* alters the balance between SM and ceramide in mouse livers

**Fig. 4:**
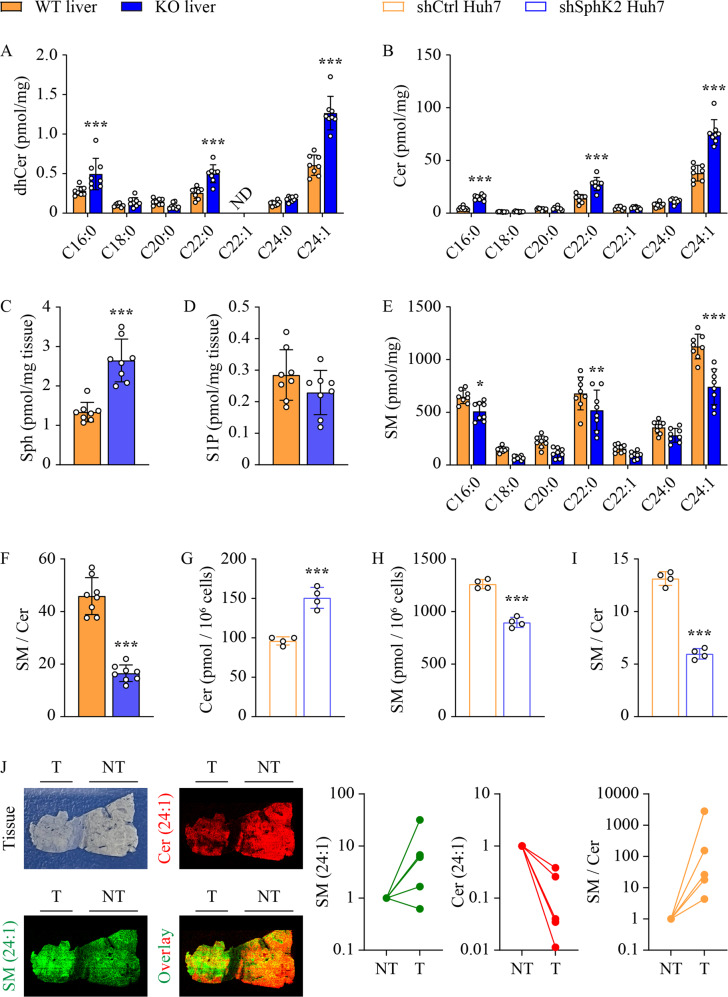
The altered sphingolipid profile in *Sphk2*-KO livers. **A**–**F** Wild-type (WT) and *Sphk2* knockout (KO) mice were fed with a high-fat, high-sugar diet (HFHSD) for 46 weeks. **G**–**I** Huh7 hepatic cells were transduced with lentiviral-based short hairpin RNA (shRNA) to knock down SphK2 and then treated with a combination of free fatty acids (FFA, 200 µM palmitate + 400 µM oleate) for 48 h. Lipids were extracted in non-tumorous liver tissues and FFA-treated cells and analyzed using lipidomics. (A) dihydro-ceramide (dhCer), **B**, **G** ceramide (Cer), **C** sphingosine (Sph), **D** sphingosine 1-phosphate (S1P), and **E**, **H** sphingomyelin (SM) were determined. **F**, **I** the ratio of SM to ceramide. **J** Levels of C24:1 SM and C24:1 Cer in human HCC (T) and adjacent non-tumorous (NT) tissues were visualized and using DESI mass spectrometry imaging coupled with ion mobility and quantified by HDI software. Data are expressed as mean ± SD. *n* = 8 **A**–**F**; *n* = 4 **G**–**I**; *n* = 5 **J**. *p < 0.05; **p < 0.01; ***p < 0.001, versus control.

To explicate the lipid metabolic basis underlying the anti-NAFLD-HCC effects of *Sphk2*-KO, we examined lipid changes in non-tumorous liver tissues using lipidomics. First, we determined 12 major FFAs. The hepatic levels of oleic acid and palmitic acid, the two most abundant FFA species, were significantly decreased in *Sphk2*-KO mice (Suppl Fig. 3A). Levels of cholesterol ester (CE), diglyceride (DG), TG, phosphatidic acid (PA), phosphatidylcholine (PC), phosphatidylethanolamine (PE), phosphatidylglycerol (PG), phosphatidylinositol (PI) and phosphatidylserine (PS) were unaltered in *Sphk2*-KO livers (Suppl Fig. 3B–D). The hepatic content of neutral lipids, including CE and TG, confirmed that mice of the two genotypes developed a similar degree of hepatic steatosis (Fig. 2B). Ablation of *Sphk2* resulted in a slight elevation of free cholesterol (FC) levels in the liver (Suppl Fig. 3B). In contrast, FC levels were significantly decreased in SphK2 knockdown hepatic cells, without prominent alterations in its subcellular localization (Suppl Fig. 4A, B). Since SphK2 is key to sphingolipid catabolism, we next focused on sphingolipids. Dihydro-sphingosine, dihydro-ceramide, ceramide, and sphingosine sit proximally upstream of SphK2-mediated sphingolipid catabolism [8]. When *Sphk2* was ablated, the three most abundant hepatic dihydro-ceramide and ceramide species (C16:0, C22:0, and C24:1), as well as dihydro-sphingosine and sphingosine, were significantly increased (Fig. 4A–C and Suppl Fig. 3E). In contrast, hepatic levels of S1P and dihydro-S1P, the products of SphK2, were unchanged in these unperfused liver tissues (Fig. 4D and Suppl Fig. 3F). In the sphingolipid metabolic network, complex sphingolipids are distal to SphK2-mediated regulation. They are normally highly abundant and cannot be altered by SphK2 manipulation. Indeed, hexosyl-ceramide (HexCer) levels were unchanged (Suppl Fig. 3G). However, unexpected results were seen at SM levels. Remarkably opposed to ceramide and dh-ceramide, C16:0, C22:0, and C24:1 SM were significantly decreased in *Sphk2*-KO livers deficiency (Fig. 4E), resulting in a 64% reduction in the ratio of SM (Fig. 4F). We also examined ceramide and SM levels in FFA-treated Huh7 cells. Consistent with the in vivo findings, knockdown of SphK2 increased ceramide and decreased SM levels, leading to a significant 55% reduction in the ratio of SM to ceramide (Fig. 4G–I). To further test the notion that SM/ceramide ratio is associated with HCC development, we examined the levels of C24:1 SM and C24:1 ceramide, the most abundant hepatic SM and ceramide species in human HCC and para-tumorous tissues, using mass spectrometry imaging. We found that C24:1 SM levels were increased, whereas C24:1 ceramide levels were decreased, in the tumorous tissues as compared with para-tumorous tissues (Fig. 4J). Notably, the SM/cer ratios were increased in the tumorous tissues of all specimens (Fig. 4J).

### Deletion of *Sphk2* results in downregulation of CERT

**Fig. 5:**
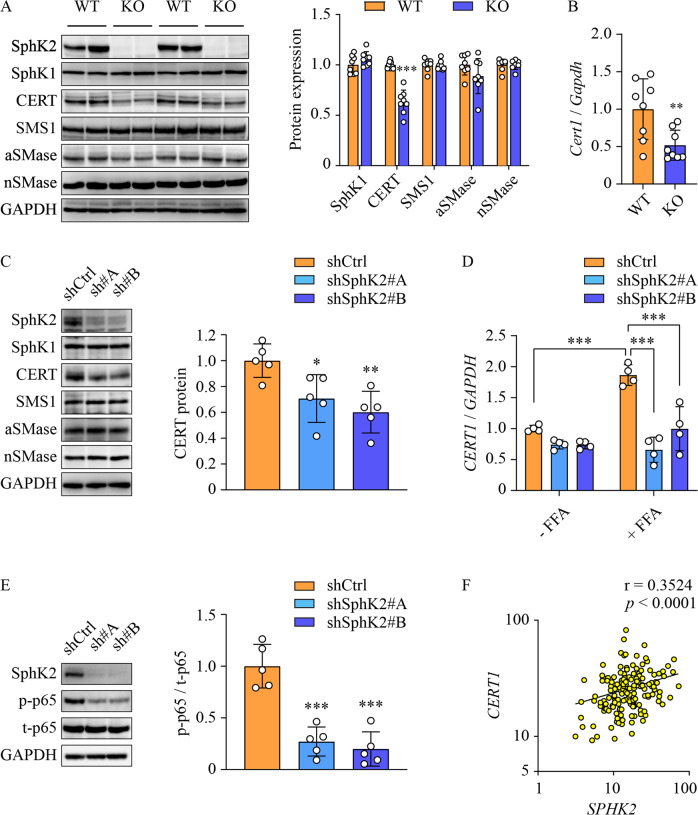
SphK2 deficiency downregulates CERT both in vivo and in vitro. **A**, **B** Wild-type (WT) and *Sphk2* knockout (KO) mice were fed with a high-fat, high-sugar diet (HFHSD) for 46 weeks. **A** Proteins were extracted from non-tumorous liver tissues and analyzed using Western blotting. Expression levels of the indicated proteins were quantified; *n* = 8. **B** mRNA expression of *Cert1*, the gene encoding mouse CERT, was examined by real-time RT-PCR; *n* = 8. **C**–**E** Huh7 hepatic cells were transduced with lentiviral-based short hairpin RNA (shRNA) to knock down SphK2 and then treated with a combination of free fatty acids (FFA, 200 µM palmitate + 400 µM oleate) for 48 h. **C**, **E** Proteins were extracted from cells and analyzed using Western blotting. The CERT protein expression and phospho-p65 over total p65 (p-p65/t-p65) were quantified; *n* = 5. **D** mRNA expression of *CERT1*, the gene encoding human CERT, was examined in the presence and absence of the FFA treatment by real-time RT-PCR; *n* = 4. **A**–**E** Data are expressed as mean ± SD. *p < 0.05; **p < 0.01; ***p < 0.001; versus control. **F** The correlation of *SPHK2* and *CERT1* mRNA expression was analyzed in human HCC subjects with body mass index > 25. Data are sourced from TCGA (https://portal.gdc.cancer.gov).

Given the importance of SM/ceramide ratio in HCC, we next investigated the cause of SM/ceramide ratio change upon SphK2 deficiency. To this end, we examined key factors that primarily regulated the interconversion between ceramide and SM in the livers of HFHSD-fed mice. These factors include CERT, SMS1, aSMase, and nSMase. Ablation of *Sphk2* led to a downregulation of CERT protein, while SMS1, aSMase and nSMase levels were unaltered (Fig. 5A). In line with this, *Cert1* mRNA was significantly decreased in *Sphk2*-KO livers (Fig. 5B). These findings were confirmed in FFA-treated Huh7 cells, in which knockdown of SphK2 resulted in a downregulation of CERT protein and mRNA (Fig. 5C, D), suggesting that SphK2 regulated CERT expression at the transcriptional level. Nuclear factor-κB (NF-κB) is a primary transcription factor of CERT [31], and thus we examined the activation of NF-κB by determining the phosphorylation of its p65 subunit. Knockdown of SphK2 greatly reduced p65 phosphorylation in FFA-treated cells (Fig. 5E), associated with the decrease of CERT protein and mRNA levels (Fig. 5C, D). In contrast, p-p65 levels were relatively low, and shSphK2 marginally repressed p65 phosphorylation, in the absence of FFA loading (Suppl Fig. 5B). In accord, shSphK2 did not alter CERT protein and mRNA expression when no FFA was added (Fig. 5D and Suppl Fig. 5A, B). We further analyzed the correlation between *SPHK2* and *CERT1* genes in the human HCC dataset sourced from TCGA, focusing on NAFLD-HCC. Due to the lack of NAFLD diagnostic information, we extracted data from human subjects with body mass index (BMI) > 25 in the TCGA-LIHC dataset, as BMI is strongly associated with NAFLD risk [30]. We found that *SPHK2* and *CERT1* mRNA levels were positively correlated in overweight or obese HCC subjects (Fig. 5F).

### Downregulation of CERT is critical for the anti-cancer effects of SphK2 deficiency

**Fig. 6:**
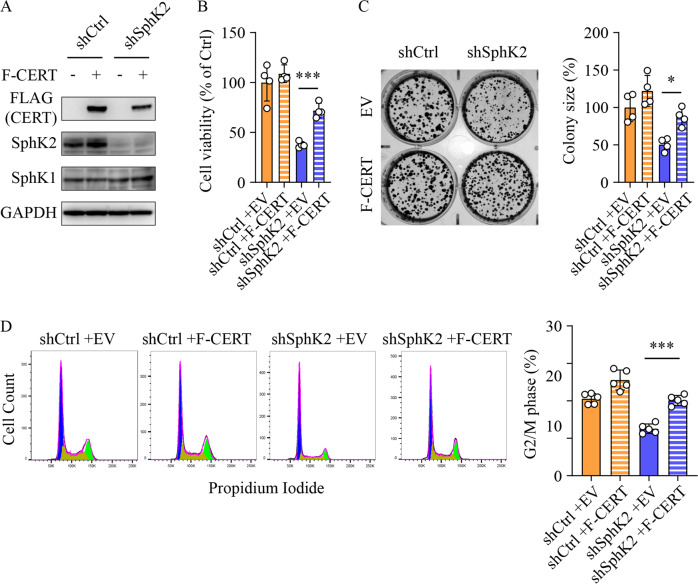
Overexpression of CERT reversed the anti-cancer effects of SphK2 deficiency. Huh7 hepatic cells were transduced with lentiviral-based short hairpin RNA (shRNA) to stably knock down SphK2 and then transiently transfected with FLAG-tagged CERT, prior to the treatment with a combination of free fatty acids (200 µM palmitate + 400 µM oleate). **A** Western blotting analyses of the indicated proteins. **B** Cell viability was determined by MTS assay in cells treated with FFA for 48 h; *n* = 4. **C** Colony formation assay was performed over ten days of cell culture in the presence of FFA. Transfection of FLAG-tagged CERT was re-performed on day 5 of the culture to maintain the CERT expression. The size of colonies was quantified; *n* = 4. **D** Cell cycle phases were examined in cells treated with FFA for 24 h, using flow cytometry with propidium iodide staining. G_0_/G_1_, S, and G_2_/M phase populations were estimated using FlowJo software and are indicated in blue, yellow, and green in histograms, respectively. The percentage of the G_2_/M phase, indicative of cell cycle progression, was analyzed; *n* = 5. Data are expressed as mean ± SD. *p < 0.05; ***p < 0.001; as indicated.

Having demonstrated that ablation of *Sphk2* simultaneously increased ceramide and decreased SM levels (Fig. 4), we examined whether the reduced hepatic cell proliferation after SphK2 knockdown was due to the ceramide and SM changes. We increased cellular ceramide levels by treatment with exogenous C6-ceramide. In response to this compound, viable cell number was decreased to a comparable extent in both shCtrl and shSphK2 cells (Suppl Fig. 6A). We next reduced cellular ceramide levels by treatment with ceramide synthase inhibitor fumonisin b1. Rather than rescuing cell proliferation in shSphK2 cells fumonisin b1 caused a further reduction in viable cell number in combination with FFA treatment (Suppl Fig. 6B). These data indicate that increased ceramide alone was not sufficient to cause the inhibition of cell proliferation in shSphK2 cells. To further explicate whether the anti-cancer effects of SphK2 deficiency could be attributed to a combinational effect of both SM and ceramide changes, we overexpressed CERT in Huh7 cells prior to FFA treatment (Fig. 6A). Although it had minor impacts on cell viability and colony size in control cells, overexpression of CERT significantly increased both of these parameters in SphK2 deficient cells (Fig. 6B, C). Consistent with this, enforced expression of CERT restored cell cycle progression to the G2/M phase in SphK2 knockdown cells to a level comparable with control cells (Fig. 6D). These data indicate that downregulation of CERT and the subsequent disruption of the SM/ceramide balance were, at least in part, responsible for anti-cancer effects of SphK2 deficiency in NAFLD-HCC.

## Discussion

The anti-cancer effects of SphK2 deficiency have been demonstrated in HCC cell lines and xenograft models [26, 27]. However, the in vivo role of SphK2 was never studied in any primary HCC models, which restricts the development of new anti-HCC treatments targeting SphK2. To define the exact role of SphK2 in NAFLD-related HCC in vivo, we fed *Sphk2*-KO mice an HFHSD to induce liver tumors. The HFHSD model recapitulates the development of spontaneous HCC in NAFLD livers and enables a focus on the underlying lipid metabolic causes. Long-term exposure to an HFHSD leads to liver tumors in mice with varied incidence rates up to 68.8% [32–35]. We identified visible liver tumors and neoplastic lesions in 35.7% of WT mice (Fig. 1J). In marked contrast, no tumors or lesions were found in *Sphk2*-KO mice (Fig. 1J), defining a requirement for SphK2 in NAFLD-HCC development in vivo. Microenvironmental changes and pro-cancer cellular alterations contribute synergistically to HCC development [36]. SphK2 deficiency abrogated HFHSD-induced body weight gain, hyperlipidemia, hepatic inflammation and fibrosis (Figs. 1 and 2), resulting in a tumor-suppressive microenvironment. Meanwhile, SphK2 deficiency inhibited hepatocyte proliferation under a high-fat condition both in vivo and in vitro (Figs. 2H and 3), which demonstrates another layer of its anti-NAFLD-HCC effects at the cellular level. In line with this, we and others have found that ablation of SphK2 impairs, whereas overexpression of SphK2 activates the signaling of Akt, a master regulator of cell proliferation [28, 29, 37]. Our results collectively indicate that SphK2 deficiency reduces HCC risk in fatty livers at both systemic and cellular levels, providing in vivo experimental evidence for clinical trials of SphK2 inhibitors as systemic therapies against NAFLD-HCC. The anti-HCC effects of SphK2 deficiency warrant further examination in more complex primary HCC mouse models, including HFHSD combined with DEN or fibrogenic agents. In addition, the hepatocyte-autonomous roles of SphK2 in HCC development should be further elucidated using cell type-specific knockout mice.

Dysregulated lipids are believed to drive NAFLD progression to HCC, but exactly which hepatic lipids impose significant HCC risk remains elusive [5]. We found that both WT and *Sphk2*-KO mice developed a similar level of hepatic steatosis upon HFHSD feeding, as reflected in H&E staining (Fig. 2A, B) and hepatic TG and CE determination (Suppl Figs. 3B and 3C). This supports the notion that accumulation of neutral lipids in the liver represents the degree of simple hepatosteatosis but not the risk for carcinogenic injury [38]. In contrast, increased FC in hepatocytes promotes cytotoxicity and proinflammatory responses, predisposing to the development of NASH and HCC [39–43]. Correspondingly, dietary cholesterol reprograms genetic and metabolic signatures in the liver, in favour of NASH-HCC [44]. Adenoviral overexpression of SphK2 significantly reduces total hepatic cholesterol (FC + CE) in HFD-fed mice [29]. Consistently, *Sphk2*-KO mice exhibited slightly elevated hepatic FC levels, with unchanged CE levels (Suppl Fig. 3B). *Sphk2*-KO profoundly downregulates gene expression of many key regulators in hepatic cholesterol homeostasis, including low-density lipoprotein receptor, sterol 27-hydroxylase, farnesoid X receptor α and bile salt export pump [45]. This might lead to hepatic cholesterol disturbance with long-term HFD feeding. Contrasting with the FC changes in vivo, SphK2 deficiency decreased FC levels in hepatic cells in vitro (Suppl Fig. 4A), implicating SphK2 in non-hepatocyte-autonomous regulation of hepatic cholesterol homeostasis in vivo. Given the importance of FC in non-alcoholic steatohepatitis (NASH) and HCC, the role of SphK2 in hepatic cholesterol homeostasis should be investigated in further studies. In the present work, this minor hepatic FC increase was not sufficient to overwhelm the tumor-suppressive effects of *Sphk2*-KO. In addition, SphK2 did not alter the hepatic levels of phospholipids (Suppl Fig. 3D). Therefore, it was unlikely that SphK2 regulated HCC development via cholesterol or phospholipid metabolism in the liver. Accumulation of palmitic acid, the most abundant saturated FFA, in the liver can induce NASH characterized by hepatic fibrosis and inflammation, leading to HCC [46, 47]. In addition, the accumulation of oleic acid, the most abundant mono-unsaturated FFA, in the liver also contributes to HCC development by promoting hepatic cell proliferation [48]. Consistent with its HCC-suppressive phenotype, *Sphk2*-KO decreased hepatic levels of both palmitic acid and oleic acid (Suppl Fig. 3A). The decrease of hepatic FFA might result from the reduction of circulating NEFA in *Sphk2*-KO mice (Fig. 1G), as the latter is the primary source of the former in human fatty liver disease [49]. The regulation of hepatic FFA levels might, at least in part, explain the anti-NAFLD-HCC effects of *Sphk2*-KO.

It was expected that ablation of *Sphk2* would cause significant sphingolipid remodelling. Indeed, dihydro-ceramide, ceramide, dihydro-sphingosine and sphingosine were increased in *Sphk2*-KO livers (Fig. 4A–C and Suppl Fig. 3E), as all of them reside upstream of SphK2-mediated enzymatic regulation in the sphingolipid metabolic network [8]. However, S1P, the catalytic product of SphK2, was only marginally decreased in *Sphk2*-KO livers (Fig. 4D). This could be due to the presence of blood in the tissue samples. S1P levels are much higher in blood than in tissues, due to the lack of S1P-degrading enzymes, S1P lyase and S1P phosphohydrolase, in erythrocytes [50]. Any blood contamination in isolated tissues would cause inaccurate quantitation of S1P [51]. This is more problematic in *Sphk2*-KO mice. SphK2 is essential for the disposal of blood S1P in the liver, and thus ablation of *Sphk2* ordinarily elevates blood S1P levels by 2-3 fold [51, 52]. We did not perfuse mice prior to the isolation of the liver tissues for this study, as it was important to examine the immune cell infiltration. Therefore, it is not surprising that S1P levels were only marginally reduced in unperfused *Sphk2*-KO livers (Fig. 4D). In support of this, we have previously demonstrated that S1P levels are significantly decreased in both *Sphk2* global KO and hepatocyte-specific KO (LKO) mouse livers after perfusion [28, 53]. SphK1 redundancy after *Sphk2* deletion might also contribute to the maintenance of hepatic S1P levels. However, SphK1 is expressed at low levels [29] and only accounts for ~10% of total SphK activity [25] in the liver. We found no changes in SphK1 levels after *Sphk2* knockout (Fig. 5A). Although the unchanged protein expression cannot preclude possible redundancy in enzymatic activity, this data suggests that SphK1 redundancy, if present, would be minor.

SM represents the most abundant sphingolipid subclass [13]. It is also an essential structural lipid in cellular membranes, particularly lipid rafts [54], where it promotes cell proliferation and growth [14, 19, 55, 56]. In marked contrast, ceramide is known as a tumor-suppressive factor in most cancer types via a variety of biological actions, including the inhibition of cancer cell proliferation [10, 24]. Consistently, we found that SM levels were increased, whereas ceramide was decreased in human HCC tissues, as compared with the para-tumorous tissue (Fig. 4J). C6-ceramide decreased cell viability (Suppl Fig. 6A), confirming the cytotoxic effects of this compound. However, the reduction of cell viability was comparable between control and SphK2 knockdown cells, indicating that increased ceramide alone could not explain the inhibition of cell proliferation induced by SphK2 deficiency (Suppl Fig. 6A). We also decreased endogenous ceramide levels by treatment with the ceramide synthase inhibitor fumonisin b1. If increased ceramide was a leading cause for the defects upon SphK2 deficiency, fumonisin b1 should restore cell proliferation in SphK2 knockdown cells. However, fumonisin b1 induced an additional decrease in cell viability by up to 15% in shSphK2 cells as compared with shCtrl cells (Suppl Fig. 6B). These data together suggest that increased ceramide was not the sole factor in SphK2 deficiency-mediated regulation. Instead, mounting evidence has demonstrated that the ratio of SM/ceramide might determine the outcome of cancer development [57]. In line with this notion, SphK2 deficiency differentially regulated ceramide and SM levels in HFHSD-fed mouse livers and FFA-treated Huh7 hepatic cells, leading to a profound decrease in SM/ceramide ratio (Fig. 4F, I), which was associated with its anti-HCC phenotype (Fig. 1).

The ratio of SM/ceramide is dictated by three key regulators: CERT, SMS, and SMases, and the key step in ceramide-to-SM conversion is ceramide transport from the endoplasmic reticulum to the *trans*-Golgi, mediated by CERT [12]. It is well demonstrated that CERT can determine the ratio of SM/ceramide, independent of SMS and SMases [12, 58–61]. *Sphk2*-KO resulted in a significant downregulation of CERT but did not alter levels of SMS1, aSMase, and nSMase in the liver tissues, indicating that the change of CERT accounted for the decreased ratio of SM/ceramide (Fig. 5A, B). This was also observed in hepatic cells treated with FFAs (Fig. 5C, D). SphK2 deficiency decreased CERT mRNA levels both in vivo and in vitro (Fig. 5B, D), indicative of transcriptional regulation. It has been demonstrated that CERT gene transcription is regulated by NF-κB, and thus tumor necrosis factor α can increase CERT mRNA levels by 3-4 fold via NF-κB activation [31]. SphK2 knockdown profoundly suppressed NF-κB activation in cells loaded with FFA (Fig. 5E). This might contribute to the downregulation of CERT. In support of this, both knockdown and inhibition of SphK2 inhibit NF-κB activation in regorafenib-resistant HCC cells [27]. Exactly how SphK2 regulates NF-κB activation and whether other transcription factors are implicated in the regulation of CERT levels are worthy of further investigation.

Multiple lines of evidence have demonstrated that CERT is highly expressed in drug-resistant human subjects with ovarian and breast cancers [60, 62, 63]. In addition, knockdown of CERT sensitizes HCT-116 human colon cancer cells, BT474, HCC1954 and SK-BR3 HER2 + breast cancer cell lines, MDA-MB-231 human triple-negative breast cancer cells and A549 human lung carcinoma cells to apoptosis induced by chemotherapeutic agents [60, 62]. Furthermore, knockdown or inhibition of CERT leads to cell cycle arrest in murine embryonic cells and paclitaxel-treated HCT-116 cells [60, 62, 64]. To examine if CERT downregulation was critical for the anti-HCC effects of SphK2 deficiency, we overexpressed CERT in control and SphK2 knockdown hepatic cells. We found that overexpression of CERT restored cell proliferation, colony formation, and cell cycle progression in SphK2-deficient hepatic cells (Fig. 6). Notably, SphK2-mediated regulation of CERT is dependent on a high-fat environment. Knockdown of SphK2 failed to downregulate CERT levels or suppress hepatic cell proliferation in the absence of FFA treatment (Fig. 5D, Suppl Figs. 2, 5A, B). The different effects of SphK2 knockdown on CERT expression and cell proliferation between untreated and FFA-treated hepatic cells might be attributed to the states of NF-κB activation (Suppl Fig. 5B). FFA treatment dramatically activated NF-κB, which was suppressed by SphK2 deficiency (Suppl Fig. 5B). In contrast, the active form of NF-κB (p-p65) stayed at a relatively low level in untreated cells (Suppl Fig. 5B). In accord, CERT protein levels were regulated in the same fashion (Suppl Fig. 5B). Similarly, NF-κB is activated in drug-resistant cancer cells [27], and CERT is highly expressed in drug-resistant human cancers [60, 62, 63]; whereas, a lower CERT expression level is reported in some human cancers in the absence of chemotherapies [59, 65, 66]. These studies suggest that CERT may play a more explicit pro-cancer role under stress, such as chemotherapies and overnutrition, when the conversion of pro-apoptotic/anti-proliferative ceramide to proliferative SM is more critical for cell proliferation and growth.

In summary, the current study provides both in vivo and in vitro evidence demonstrating a pro-cancer role of SphK2 in the development of NAFLD-HCC (Suppl Fig. 7). Knockout of *Sphk2* suppressed HFHSD-induced HCC in mice, associated with inhibition of hepatic cell proliferation in a tumor-suppressive microenvironment. Mechanistically, SphK2 deficiency resulted in downregulation of CERT, leading to a reduced ratio of SM/ceramide, which is unfavourable for HCC cell proliferation. Restoration of CERT expression substantially improved hepatic cell proliferation in SphK2 deficient cells. Our findings demonstrate the SphK2/CERT axis as a novel therapeutic target for NAFLD-HCC, although the intricate role of these coupled lipid regulators in different cancer contexts warrants further investigation.

## Materials and methods

### Animals

WT and *Sphk2*-KO mice on a C57BL/6 J background were used according to protocols (#2019-033) approved by Research Ethics and Governance Office, Royal Prince Alfred Hospital, Sydney, Australia. *Sphk2*-KO mice were obtained from Dr Richard Proia, National Institutes of Health, USA [67]. Mice were housed in a temperature-controlled, pathogen-free environment on a 12-hour light/dark cycle, and allowed food and water *ad libitum*. No statistical methods were used to predetermine the sample size. The sample size was estimated based on similar published studies indicating an up to 68.8% incidence of HCC tumors after HFD feeding [32–35]. We adopted a sample size of *n* = 14 per group, which was sufficient to achieve statistically significant differences. Male mice aged 8 weeks with matched body weight were fed with an HFHSD, using the recipe detailed in [68] with no cholesterol, for 46 weeks. Liver tissue and plasma were collected after 16 h starvation at the endpoint of the project. Levels of plasma NEFA, TC, TG, and ALT activity were analyzed using colorimetric assays (NEFA, TC, and TG kits from Fujifilm Wako Diagnostics, Osaka, Japan; ALT kit from Sigma-Aldrich, St. Louis, USA), as described previously [69].

### Histological and immunohistochemical staining

The histology of mouse liver tissues was examined by H&E (Sigma-Aldrich, St. Louis, USA) staining. The grading of NAS, encompassing steatosis, ballooning, and inflammation scores, was determined by three experienced researchers who were blinded to the experimental groups based on H&E staining following the scoring system established by Liang et al. [70]. Liver fibrosis was examined by Picro Sirius Red (PSR) staining (Sigma-Aldrich, St. Louis, USA). Immunohistochemical staining of Ki67 was performed with Ki67 antiserum (Abcam #ab15580, Cambridge, UK). Images were taken using a Nikon NiE microscope and quantified using ImageJ software (FIJI version 1.52).

### Cell culture

The Huh7 hepatic cell line was obtained from and authenticated (Dec 2021) by CellBank Australia. Cells were tested mycoplasma-free by MycoAlert Detection Kit (Lonza Bioscience, Basel, Switzerland). Cells were maintained in Eagle’s minimal essential medium (DMEM) supplemented with 10% v/v fetal calf serum and 100 U/ml penicillin/streptomycin at 37 °C in a humidified incubator. Flag-tagged human CERT plasmid in pcDNA3.1/Zeo(+) vector was obtained from Genscript (Galaxis West Lobby, Singapore). Plasmid transfection was conducted using lipofectamine LTX PLUS reagent (Thermo Fisher, Waltham, USA). Short-hairpin RNAs (shRNAs) targeting SphK2 (#A, TRCN0000036973 and #B, TRCN0000359275) were constructed in pLKO.1 lentiviral vector (Sigma-Aldrich, St. Louis, USA). The lentivirus was generated in HEK293T cells using plasmids gifted from Dr. Didier Trono through Addgene, including pMD2.G, pMDLg/pRRE and pRSV-Rev [71]. Lentiviral transduction was carried out as described previously [28]. To simulate a high-fat environment, we treated cells with a combination of bovine serum albumin-coupled palmitate (200 µM, Sigma-Aldrich, St. Louis, USA) and oleate (400 µM, Sigma-Aldrich, St. Louis, USA). C6-ceramide and fumonisin b1 were supplied by Avanti Polar Lipids (Alabaster, USA) and Cayman Chemical (Michigan, USA), respectively.

### Cell viability assay

Cell viability was examined by 3-(4,5-dimethyl-thiazol-2-yl)-5-(3-carboxymethoxyphenyl)-2-(4-sulfophenyl)-2H-tetrazolium, inner salt (MTS) assay (Promega, Madison, USA). The luminescence was determined at 490 nm on a TECAN Infinite M1000Pro plate reader.

### Colony formation assay

Huh7 cells were seeded at 400 (Fig. 3B) or 800 (Fig. 6C) cells/well in 6-well plates. Cells were treated with FFA combination for 10 days. Colonies were fixed with 4% cold paraformaldehyde, followed by crystal violet (0.5% w/v, Sigma-Aldrich, St. Louis, USA) staining [72]. Images were captured using a ChemiDoc^TM^ Touch Imaging System (Bio-Rad Laboratories, Hercules, USA) and quantified using ImageJ software (FIJI version 1.52).

### Flow cytometry

Cell cycle was examined in Huh7 cells fixed with 70% cold ethanol at 4 °C overnight. The fixed cells were then incubated with FxCycle™PI/RNase Staining Solution (Thermo Fisher, Waltham, USA) for 30 min at room temperature and analyzed using BD LSRII (BD Biosciences, Franklin Lakes, USA) [72]. G2/M phase was determined using FlowJo version 10 (BD Biosciences, Franklin Lakes, USA) in Waston (Pragmatic) mode.

### Confocal microscopy

Intracellular FC was probed by the transfection of mCherry-D4H [73, 74], while the nuclei were counterstained with ProLong Glass Antifade Mountant with NucBlue Stain. The confocal microscopy was performed using a Nikon C2 microscope, and the images were processed using FIJI ImageJ software.

### Lipidomics

Lipids were extracted from mouse liver tissues or Huh7 cells in 1 mL methanol containing a cohort of internal lipid standards (Avanti^®^ Polar Lipids, Alabaster, USA). The liver tissues isolated from the first 8 animals of each genotype were analysed. Hepatic levels of TG, CE, and phospholipids were quantified using untargeted lipidomic profiling on a Q Exactive HF-X mass spectrometer, following lipid separation on a Waters Acquity C18 UPLC column [53]. LipidSearch software was used for lipid annotation, chromatogram alignment and peak integration [75]. In contrast, FFAs, DG, FC, and sphingolipids were determined by targeted lipidomics on a TSQ Altis triple quadrupole mass spectrometer, following lipid separation on an Agilent Eclipse Plus C8 column [28, 53]. Peaks were integrated using Xcalibur (Thermo Fisher, Waltham, USA) [28].

### Mass spectrometry imaging

Human specimens encompassing both HCC and para-tumorous tissues were obtained from Royal Prince Alfred Hospital, Sydney, Australia, according to protocols (#2019/ETH13790) approved by the Sydney Local Health District Human Research Ethics Committee. Informed consent was obtained from all subjects. The specimens were fixed in 4% paraformaldehyde and cut into 40 µm-thick cryo-sections without embedding in paraffin or OCT. Lipid imaging was performed with a Prosolia 2D Desorption Electrospray Ionisation stage and a Synapt G2-Si QToF mass spectrometer with ion mobility using high and low fragmentation energy, denoted as DESI-HDMS^E^. The DESI solvent was 98:2 methanol:water, supplemented with 0.01% formic acid and 200 pg/µL Leu-enkephalin, and delivered at a flow rate of 2 µL/min. DESI imaging data were acquired using MassLynx (Waters, Milford, USA), and ion mobility drift time was processed with High Definition Imaging (HDI) software (Waters, Milford, USA) to calculate collision cross section (CCS) for lipid identification [76]. To verify lipid identifications, we scanned synthetic d18:1/24:1 ceramide and d18:1/24:1 sphingomyelin pure compounds (Sigma-Aldrich, St. Louis, USA) to confirm their mass and drift times. The obtained masses were compared against the LipidMaps database and the unified experimental CCS database. The signal intensities of these two lipids in tumor and non-tumor areas were analyzed using HDI software.

### Western blotting

Proteins were extracted from liver tissue (isolated from the first 8 animals of each genotype) and Huh7 cells. Immunoblotting was conducted with the following antibodies: SphK1 (#12071), SphK2 (#32346, human-specific, used for Huh7 cells), FLAG (#14793), p-p65 (#3033), t-p65 (#4764) and GAPDH (#5174) from Cell Signaling Technology (Danvers, USA); nSMase (#ab131330) from Abcam (Cambridge, UK); SphK2 (#17096-1-AP, used for mouse liver tissues), CERT (#15191-1-AP), SMS1 (#19050-1-AP) and aSMase (#14609-1-AP) from Proteintech (Rosemont, USA). Chemiluminescence was detected with a BIORAD ChemiDoc TOUCH imaging system.

### Quantitative RT-PCR

RNA extraction and reverse transcription were performed, as previously described [75]. RT-PCR was conducted on a Roche Lightcycler 480 machine using SensiFAST™ SYBR Lo-ROX Kit (Bioline, London, UK). Primer sets used for PCR were human/mouse *CERT1/Cert1* F-*CGATGTGTCCGTGCCAAAAT*, R-*CCATCCTCCAGGGTTCACATT* (designed using Primer-BLAST, National Center for Biotechnology Information, US); mouse *Gapdh* F-*GCCTGGAGAAACCTGCCAAG*, R-*TCATTGTCATACCAGGAAATG* [77]; human *GAPDH* F-*CGGAGTCAACGGATTTGGTC*, R-*CCATGGGTGGAATCATATTGG* [78].

### Gene correlation analysis

mRNA expression data of *SPHK2* and *CERT1* were extracted from the Liver Hepatocellular Carcinoma (LIHC) dataset generated by TCGA via https://portal.gdc.cancer.gov. We assessed 158 primary liver cancer cases that were overweight/obese subjects (BMI > 25). After determining both variables were not normally distributed by the Shapiro-Wilk test, *SPHK2* and *CERT1* data (transcripts per million) were log-transformed (natural log), followed by Pearson’s correlation analyses using Prism 9 (GraphPad, version 9).

### Statistics

Comparisons between two groups were analyzed by unpaired two-tailed *t*-tests, multiple comparisons were analyzed by one-way ANOVA with Tukey tests, and variance between groups were analyzed by F test, using GraphPad Prism version 9. The exact sample size and number of replicates (*n*) are indicated in the figure legends. Differences at values of *p* < 0.05 were considered significant.

## Supplementary information

Supplemental Figures

## Data Availability

All data supporting the findings of this study will be made available by the authors without undue reservation.
