# Supplementary material for: Ablation of sphingosine kinase 2 suppresses fatty liver-associated hepatocellular carcinoma via downregulation of ceramide transfer protein
Source: Oncogenesis. 2022 Nov 4;11(1):67. doi: 10.1038/s41389-022-00444-0 (PMC9636415; doi:10.1038/s41389-022-00444-0)
Supplement: Supplementary file 1 — Supplemental Figures [file 41389_2022_444_MOESM1_ESM.docx]

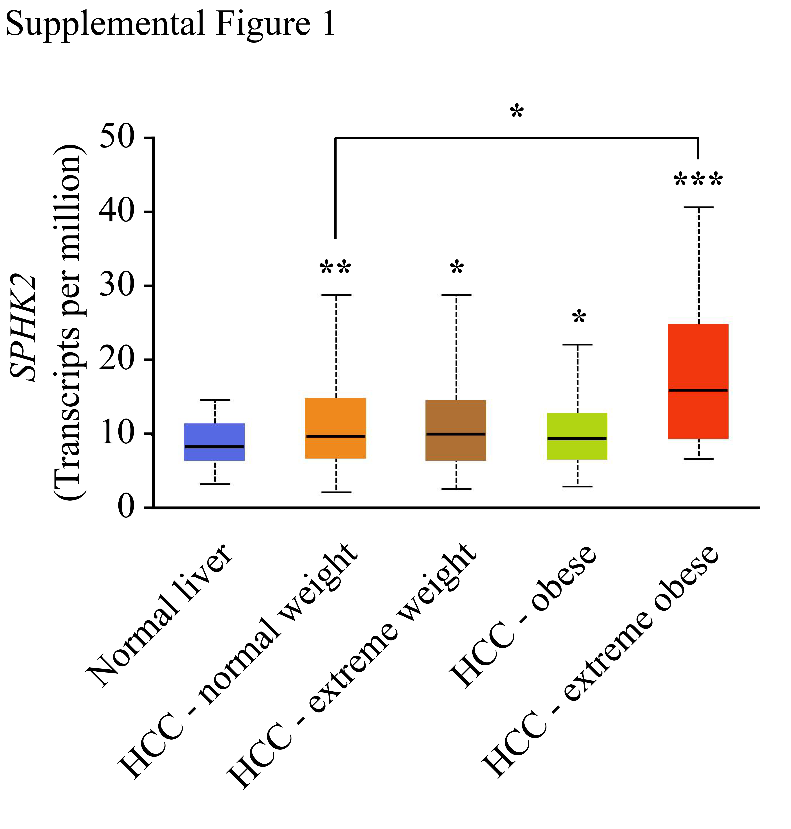


**Supplemental Figure 1. *SPHK2* expression level in human HCC.** Data are sourced from TCGA ([*https://portal.gdc.cancer.gov*](https://portal.gdc.cancer.gov)). The graph was adapted from the UALCAN platform ([*http://ualcan.path.uab.edu*](http://ualcan.path.uab.edu)) and edited in Adobe Illustrator. *, *p*<0.05; **, *p*<0.01; ***, *p*<0.001, versus normal liver, if not specified.


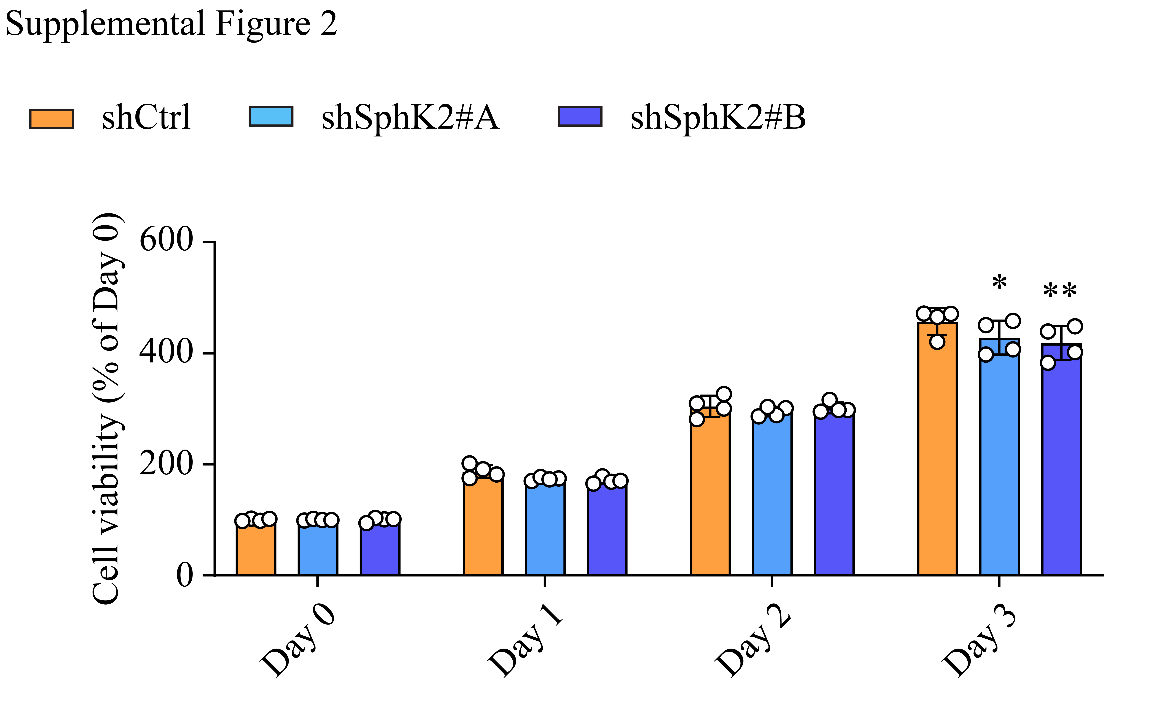


**Supplemental Figure 2. SphK2 deficiency causes a slight reduction in viable cell number in the absence of FFA.** Huh7 hepatic cells were transduced with lentiviral-based short hairpin RNA (shRNA) to knock down SphK2. Viable cell number was determined by MTS assay in cells for the indicated times; n=4. Data are expressed as mean±SD. *, *p*<0.05; **, *p*<0.01, versus shCtrl.


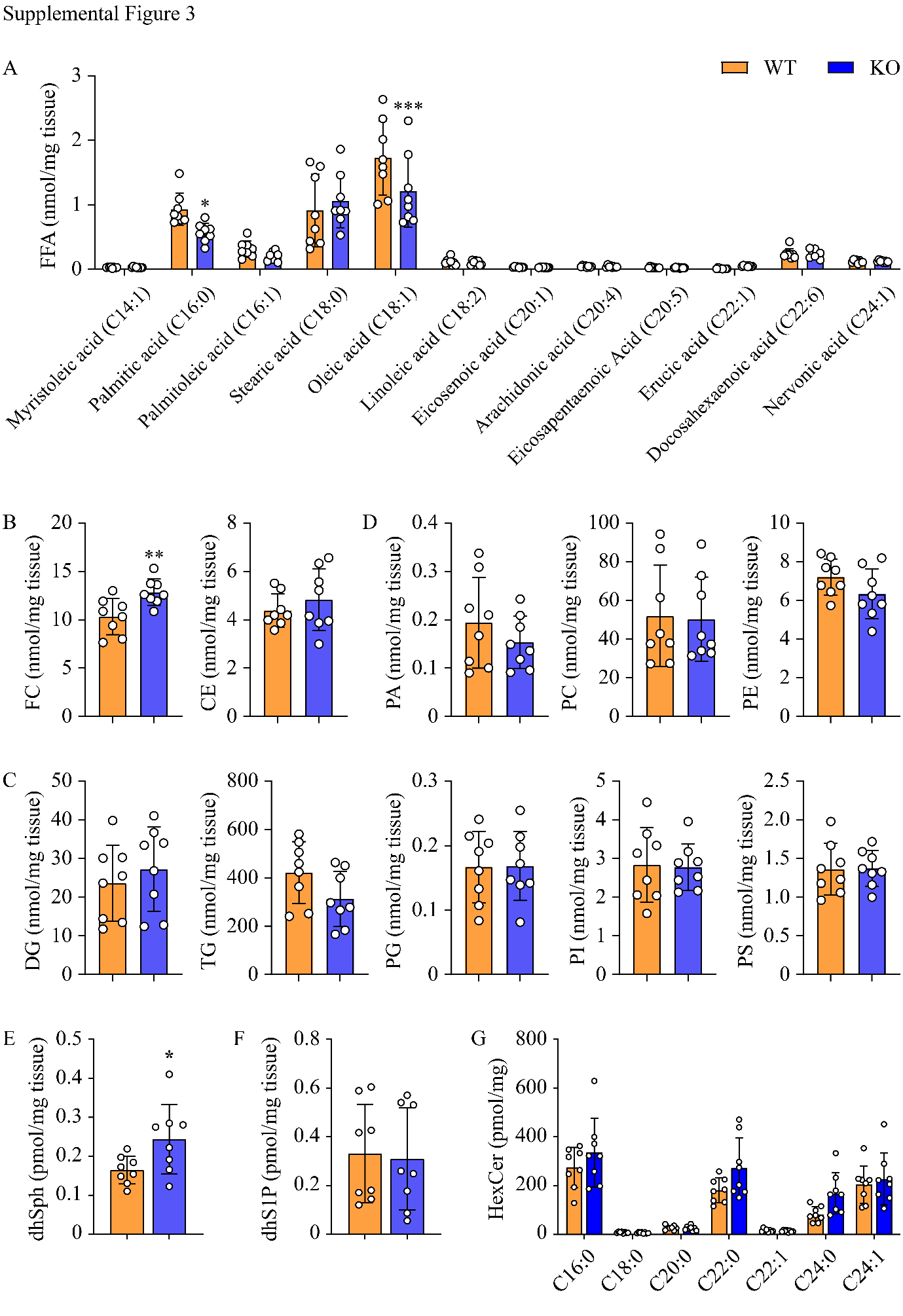


**Supplemental Figure 3. Lipid profiles in WT and *Sphk2*-KO livers.** Wild-type (WT) and *Sphk2* knockout (KO) mice were fed with a high-fat, high-sugar diet (HFHSD) for 46 weeks. Lipids were extracted from non-tumorous liver tissues and analyzed using lipidomics. (A) free fatty acids (FFA), (B) free cholesterol (FC) and cholesterol ester (CE), (C) diglyceride (DG) and triglyceride (TG), (D) phosphatidic acid (PA), phosphatidylcholine (PC), phosphatidylethanolamine (PE), phosphatidylglycerol (PG), phosphatidylinositol (PI) and phosphatidylserine (PS), (E) dihydro-sphingosine (dhSph), (F) dihydro-sphingosine 1-phosphate (dhS1P) and (G) hexosylceramide (HexCer) were determined. Data are expressed as mean±SD. n=8. *, *p*<0.05; **, *p*<0.01; ***, *p*<0.001, versus WT.


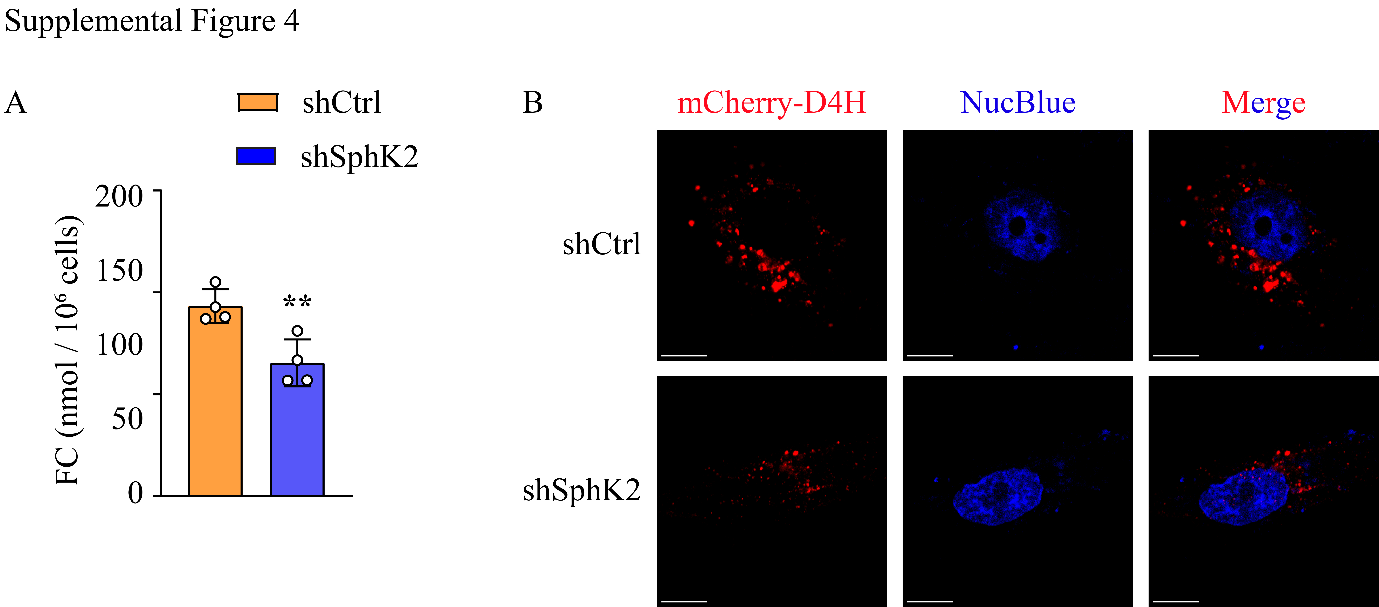


**Supplemental Figure 4. Intracellular free cholesterol content and distribution in SphK2 deficient hepatic cells.** Huh7 hepatic cells were transduced with lentiviral-based short hairpin RNA (shRNA) to knock down SphK2. (A) Lipids were extracted, and free cholesterol (FC) levels were analyzed using lipidomics. n=4. Data are expressed as mean±SD. **, *p*<0.01, versus shCtrl. (B) Intracellular FC was probed by the transfection of mCherry-D4H (shown in red), while the nuclei were counterstained using NucBlue (shown in blue). Representative images were captured using confocal microscopy; Bar = 10 μm.


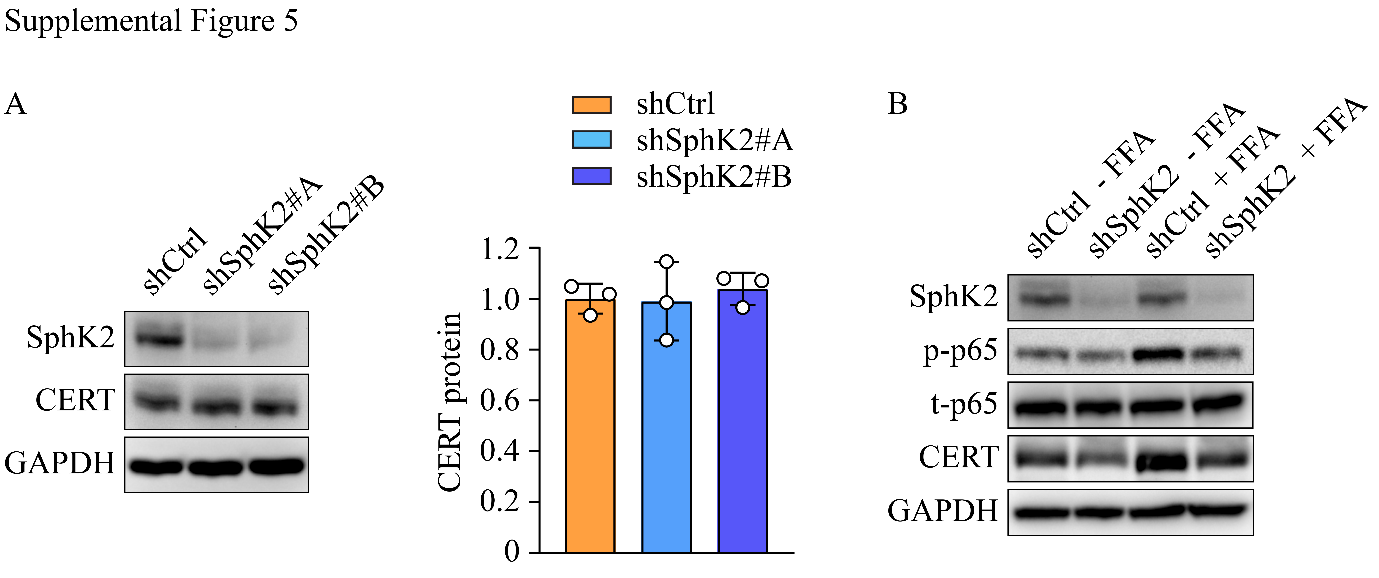


**Supplemental Figure 5. SphK2 deficiency fails to regulate CERT expression in the absence of FFA.** Huh7 cells were transduced with shRNA against SphK2. Cells were untreated (A, n=3) or treated (A, B) with a combination of free fatty acids (FFA, 200 µM palmitate + 400 µM oleate) for 48 h. Indicated proteins were analyzed using Western blotting. Data are expressed as mean±SD.


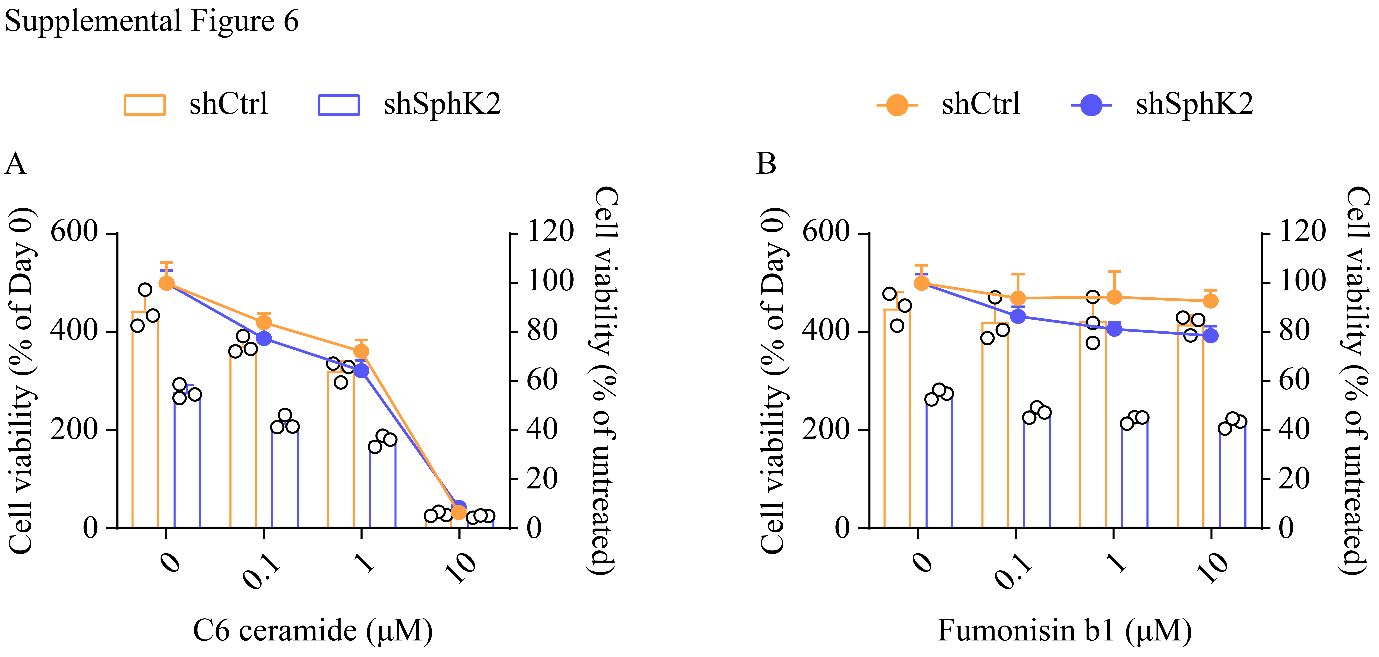


**Supplemental Figure 6. SphK2 deficiency-mediated inhibition of cell proliferation is not determined by ceramide levels alone.** Huh7 cells were transduced with shRNA against SphK2. Cells were then treated with free fatty acids (FFA, 200 µM palmitate + 400 µM oleate) for two days in combination with C6 ceramide (A) or fumonisin b1 (B) at the indicated concentrations; n=3. Data are expressed as % of viable cells before the FFA and C6 ceramide/fumonisin b1 co-treatment (Day 0) in the bar chart and as % of viable cells on Day2 relative to untreated control cells in the line chart.


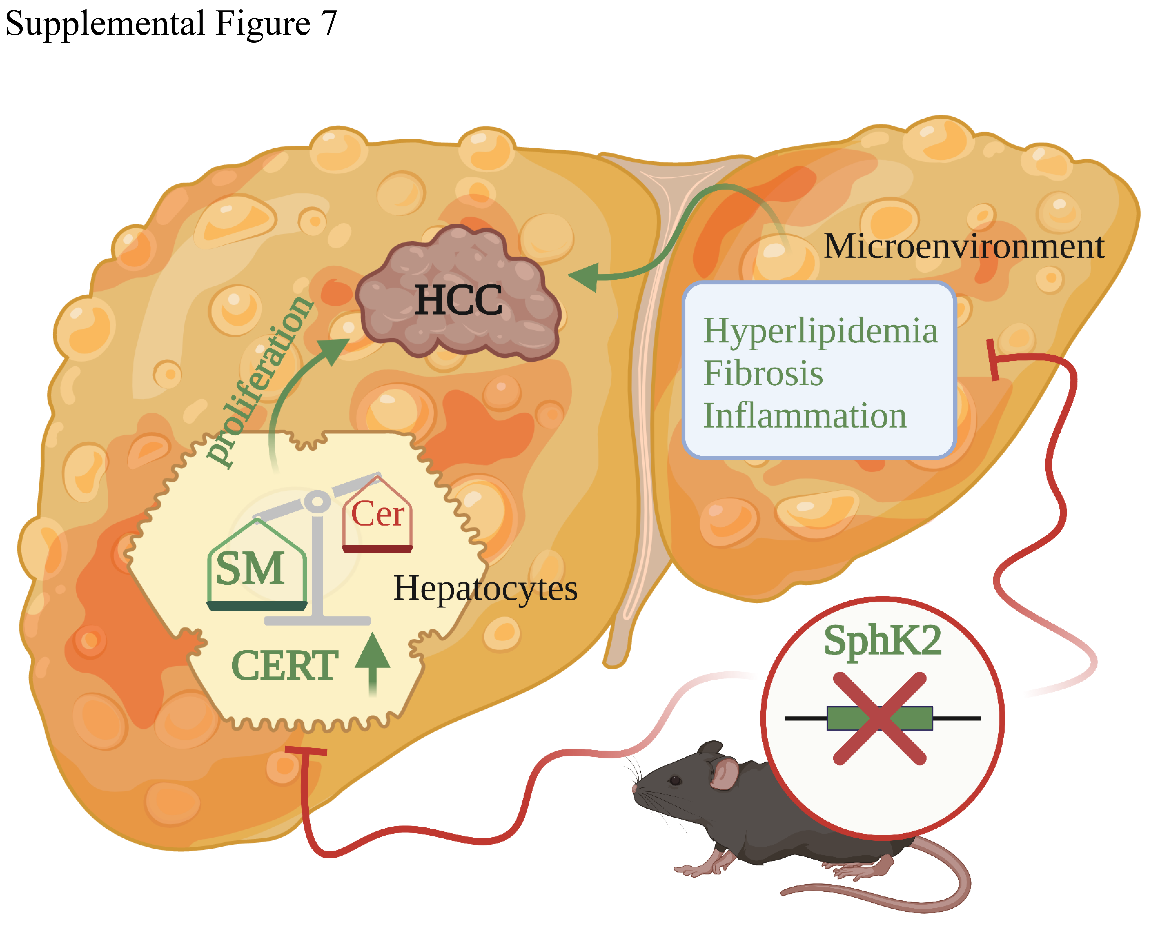


**Supplemental Figure 7. Model depicting the role of SphK2 in NAFLD-HCC.** Knockout of *Sphk2* suppresses NAFLD-HCC development in mice, which is associated with inhibition of hepatocyte proliferation in a tumor-suppressive microenvironment. This is, at least in part, attributed to a disruption of the balance between pro-cancer sphingomyelin (SM) and anti-cancer ceramide (Cer) via the downregulation of CERT. Pro-cancer factors and actions are shown in green, while anti-cancer factors and actions are shown in red. The graphical abstract was created with BioRender.com.
